# Decoding the molecular mechanism via systems biology-based insights into neoschaftoside from *Ailanthus altissima* targeting lung cancer

**DOI:** 10.1038/s41598-025-33214-0

**Published:** 2025-12-25

**Authors:** Sachin Gudasi, Dileep Kumar, Shashank Tewari, Rohini S. Kavalapure, Shriram D. Ranade

**Affiliations:** 1 Department of Pharmacognosy, KLE College of Pharmacy, KLE Academy of Higher Education and Research, Belagavi, Karnataka 590010 India; 2 Department of Pharmaceutical Chemistry, Manipal College of Pharmaceutical Sciences, Manipal Academy of Higher Education, Manipal, Karnataka 576104 India; 3 Department of Pharmaceutical Chemistry, KLE College of Pharmacy, KLE Academy of Higher Education and Research, Belagavi, Karnataka 590010 India

**Keywords:** *Ailanthus altissima*, EGFR, Lung cancer, PCA, Network pharmacology, Cancer, Computational biology and bioinformatics, Drug discovery, Oncology

## Abstract

The epidermal growth factor receptor (EGFR) is a critical regulator of multiple oncogenic signaling cascades, including MAPK, PI3K/AKT/mTOR, and JAK–STAT pathways, which collectively contribute to enhanced proliferation, angiogenesis, and resistance to apoptosis in cancer. Aberrant EGFR activation has been strongly associated with tumor progression and therapeutic resistance, underscoring its importance as a molecular target for anticancer interventions. *Ailanthus altissima* (*A. altissima*), is known for its broad-spectrum anticancer potential although the underlying mechanism has not yet been clearly defined. Therefore, in present study, bioactive constituents of *A. altissima* were systematically analyzed for their ability to modulate proteins implicated in cancer pathogenesis and subsequently overlapped with hub genes differentially expressed across cancer grades. The common targets were mapped to lung cancer associated signaling pathways, revealing EGFR as a highly modulated node. Among the identified metabolites, neoschaftoside emerged as the top-ranked bioactive interacting with EGFR-related hub genes. Later, Molecular docking and molecular dynamics (MD) simulations demonstrated strong binding affinity and conformational stability of the EGFR neoschaftoside complex. Post-MD analyses, including principal component analysis (PCA) and dynamic cross-correlation matrix (DCCM) analysis, further indicated restricted global motions and enhanced correlated dynamics, confirming structural stabilization upon ligand binding. Collectively, these findings suggest that neoschaftoside may function as a promising lead compound capable of inhibiting EGFR activity through modulation of EGFR signaling, thereby suppressing oncogenic progression.

**Supplementary Information:**

The online version contains supplementary material available at 10.1038/s41598-025-33214-0.

## Introduction

Lung cancer continues to pose a substantial global health burden, representing the foremost cause of cancer-related mortality worldwide^1^. According to the Global Cancer Observatory (GLOBOCAN) 2020 data, approximately 2.2 million new cases of lung cancer and 1.8 million associated deaths were reported, underscoring the aggressive nature and high fatality rate of this malignancy^2^. Lung cancer is clinically categorized into two main groups, including small cell lung cancer (SCLC) and non-small cell lung cancer (NSCLC). Among its histological subtypes, non-small cell lung cancer (NSCLC) accounts for approximately 85% of all lung cancer cases^3^. Despite advances in diagnostic and therapeutic strategies, the prognosis for advanced-stage NSCLC remains poor, according to American cancer society five-year survival rate of lung cancer is less than 28%. This has necessitated the continued search for more effective and durable therapeutic interventions (https://www.cancer.org/cancer/types/lung-cancer/).

Conventional treatment regimens for lung cancer include chemotherapy, targeted therapy, immunotherapy, and radiation, often used in combination based on disease stage and molecular profiling^5^. Platinum-based chemotherapeutic agents such as cisplatin and carboplatin remain the cornerstone of first-line treatment^6^. These agents form DNA cross-links and induce apoptosis through the activation of p53 and other DNA damage response pathways^7^. However, they are often accompanied by severe adverse effects including nephrotoxicity, myelosuppression, neurotoxicity, and the development of chemoresistance mediated by enhanced DNA repair, anti-apoptotic signaling (BCL2), and drug efflux pumps (MDR1).

The advent of molecularly targeted therapies has significantly improved outcomes for subsets of patients with defined oncogenic driver mutations. For instance, EGFR tyrosine kinase inhibitors (TKIs) such as gefitinib, erlotinib, and osimertinib are effective in NSCLC patients harboring EGFR mutations, by selectively inhibiting aberrant EGFR signaling, which is implicated in uncontrolled proliferation, survival, and metastasis^8,9^. Similarly, patients with ALK or ROS1 gene rearrangements benefit from ALK inhibitors (*crizotinib*, *alectinib*, *lorlatinib*), which block constitutively active fusion kinases that drive tumorigenesis^10^. Despite initial responses, the emergence of secondary mutations (EGFR T790M or ALK G1202R) and bypass signaling *via* MET or HER2 activation often leads to therapeutic resistance^11^.

A key molecular driver in NSCLC pathogenesis is the Epidermal Growth Factor Receptor (EGFR), a transmembrane receptor tyrosine kinase that plays a pivotal role in regulating cell proliferation, survival, angiogenesis, and metastasis through the activation of multiple downstream signaling cascades, including the PI3K/Akt, RAS/RAF/MEK/ERK, and JAK/STAT pathways^12,13^. Mutations in the EGFR gene, particularly exon 19 deletions and the L858R point mutation in exon 21, are frequently observed in NSCLC patients, especially in non-smokers and individuals of East Asian descent^14^. These mutations confer constitutive activation of the receptor, thereby driving tumor progression and therapeutic resistance.

The introduction and clinical use of EGFR tyrosine kinase inhibitors (TKIs) such as gefitinib, erlotinib, afatinib, and osimertinib have significantly improved progression-free survival and quality of life in patients with EGFR-mutant NSCLC^15^. These agents block the ATP-binding site of the EGFR tyrosine kinase domain, thereby inhibiting receptor autophosphorylation and downstream oncogenic signaling^8,16^. However, resistance frequently develops, with the T790M mutation—enhancing ATP affinity and reducing TKI binding responsible for 50–60% of resistance to first- and second-generation TKIs^9^. Although osimertinib was designed to target T790M, resistance still occurs, often driven by mutations such as C797S, MET amplification, or histologic transformation^17^. These limitations have spurred interest in alternative or adjunct therapies that offer improved safety and efficacy, and the potential to overcome or delay resistance.

In recent years, natural products and phytochemicals have garnered substantial interest in cancer therapeutics due to their structural diversity, multi-targeting capabilities, and favorable safety margins^18^. One such candidate is *Ailanthus altissima* (Tree of Heaven), a traditional medicinal plant used in Chinese medicine, which has been reported to possess diverse pharmacological properties including anti-inflammatory, antimicrobial, and anticancer activities^19,20^. Preliminary studies have demonstrated that *A. altissima* contains numerous bioactive compounds with potential anticancer activity, although their mechanistic roles in modulating resistance pathways in NSCLC, particularly those involving EGFR, remain largely unexplored^21^. To address this gap, the present study employs an integrated systems pharmacology approach, combining network pharmacology, molecular docking, and molecular dynamics simulation to systematically investigate the therapeutic potential of *A. altissima* against EGFR TKI resistance in lung cancer. By identifying and validating the interactions between phytoconstituents of *A. altissima* and key molecular targets implicated in lung cancer pathways, this study aims to elucidate its multi-targeted mechanisms of action and provide a scientific foundation for its development as a complementary therapeutic agent in the treatment of drug-resistant NSCLC.

## Methods

### Retrieval of bioactives

The Collection of bioactives present in *A. altissima* was identified by using published articles and Herb 2.0 (A high-throughput experiment- and reference-guided database of traditional Chinese medicine). Herb id of *A. altissima was* HERB000838. The names of the compounds collected from the herb 2.0 database were entered into the PubChem database (https://pubchem.ncbi.nlm.nih.gov/) and the SMILEs (Simplified Molecular Input Line Entry System) of the corresponding compounds were downloaded for the prediction of target genes and molecular docking.

### Target prediction of *A. altissima* compounds and lung cancer association

The SMILES representations of bioactive compounds derived from *A. altissima* were submitted to the SwissTargetPrediction platform (http://www.swisstargetprediction.ch/) to predict potential human protein targets. The resulting datasets were exported in CSV format, and compound-specific targets were curated and consolidated using Microsoft Excel. To ensure consistency, all predicted protein targets were mapped to their corresponding official gene symbols. In parallel, lung cancer-related genes were retrieved from the GeneCards database (https://www.genecards.org/) using the keyword “lung cancer” Common targets between *A. altissima* compound predictions and lung cancer-associated genes were identified through Venny 2.1.0 (https://bioinfogp.cnb.csic.es/tools/venny/), facilitating the selection of intersecting targets potentially involved in the therapeutic modulation of lung cancer^22^.

### Protein-protein interaction and MCODE cluster analysis

The common targets between *A. altissima* and lung cancer were subjected to STRING *ver. 11.5* (https://string-db.org/*)* to construct a protein-protein interaction network. The PPI data were subsequently exported into Cytoscape 3.10.1. (accessed on 27 July 2025) for further analysis of centrality measures. In the PPI, nodes represent the target proteins, while edges represent the interactions between targets. Isolated targets were excluded from this study, and the PPI network was constructed with a confidence score threshold of 0.04. Cluster analysis was conducted by using the MCODE (Molecular Complex Detection) clustering module^23^.

### Network construction and analysis

The network connecting the compounds with their respective targets and pathways was constructed by using Cytoscape 3.10.2. The interactions between the compounds and lung cancer targets were visualized. The network was then analyzed using the “Network Analyzer” tool, treating it as a direct command, and prioritizing edge count from high too low for modulation and also network analysis was performed on the basis of “*Node degree distribution*” and “*Betweenness by degree*” where parameters like eccentricity, neighbourhood connectivity and in-degree distribution were analyzed. In this network construction, nodes represent the compounds, targets, and pathways, while edges denote the interactions among these factors. Which represented the relationship between the compounds and disease targets. The degree value of interactions was utilized to judge the importance of nodes in each given network^24^.

### Gene ontology and KEGG pathway enrichment analyses

Gene Ontology (GO) and Kyoto Encyclopedia of Genes and Genomes (KEGG) pathway enrichment analyses were conducted to systematically explore the functional significance and biological pathways associated with the selected gene set. GO analysis was categorized into three main domains: Biological Process (BP), Cellular Component (CC), and Molecular Function (MF), facilitating the classification of genes based on their roles in physiological processes, subcellular localization, and molecular functions. KEGG pathway enrichment analysis was utilized to identify key signaling and metabolic pathways significantly associated with the gene list, providing deeper insights into the underlying mechanisms of action. Additionally, ClueGO and CluePedia, plugins for Cytoscape, were employed to visualize and interpret functionally grouped GO terms, particularly focusing on immune-related biological processes^25^.

### Acquisition of hub gene expression data in lung cancer

The expression profiles of the identified hub genes in different histological grades of lung cancer, including Lung Adenocarcinoma (LUAD) and Lung Squamous Cell Carcinoma (LUSC), were retrieved using the GEPIA2 (Gene Expression Profiling Interactive Analysis) database. GEPIA2 integrates RNA sequencing data from The Cancer Genome Atlas (TCGA) and the Genotype-Tissue Expression (GTEx) projects, processed through a unified analysis pipeline to ensure consistency and reliability. The dataset comprised 483 LUAD tumor samples and 347 corresponding normal lung tissues, as well as 486 LUSC tumor samples and 338 normal controls. Differential gene expression analysis was conducted to compare tumor and normal tissues, while survival analysis was performed to evaluate the prognostic significance of the hub genes. This analysis provided comprehensive insights into the expression patterns of the hub genes across different tumor grades, contributing to a better understanding of their roles in lung cancer progression and patient outcomes^26^.

### Molecular docking

The crystal structure of the EGFR tyrosine kinase binding domain (PDB ID: 4ZAU) was retrieved from the Protein Data Bank (https://www.rcsb.org/). Molecular docking studies were performed using Schrödinger’s Small Molecule Drug Discovery Suite 2023-1 (Schrödinger, LLC, New York, NY).

#### Ligand preparation

The 2D chemical structures of bioactives were sketched in Maestro and then prepared using LigPrep. This included the generation of possible tautomers and stereoisomers, correction of chiralities, and energy minimization using the OPLS4 force field. Ligands were ionised at pH 7.0 ± 0.5 using Epik.

#### Protein preparation

The protein structure was prepared using the Protein Preparation Workflow in Maestro. The procedure included the addition of missing hydrogen atoms, assignment of proper bond orders, correction of mislabeled atoms, and generation of disulfide bonds. Water molecules beyond 5 Å from any heteroatom were removed. Protonation states of ionizable residues were assigned at pH 7.0 using Epik. The H-bonding network was optimised, and a restrained minimization of the entire protein was conducted using the OPLS4 force field until the root mean square de*via*tion (RMSD) of the heavy atoms converged to 0.3 Å.

#### Grid generation

The EGFR protein possesses two lobes of kinase structure (NH_2_) N-lobe and (COOH), C-lobe, which serves as the ATP/erlotinib (cocrystal) binding region. The N-lobe is formed by the β-strand along with the one α-helical (αC) structure and the larger C-lobe is composed of α helix. The two lobes are divided by a cleft analogous to those where ATP, ATP analogues, and ATP-competitive inhibitors have been seen to bind. To define the binding site, a receptor grid was generated using the sitemap module in Schrödinger’s Glide suite.

#### Molecular docking

Molecular docking of the prepared ligands was carried out using the Glide module in Extra Precision (XP) modes. During the docking process, the van der Waals radii of nonpolar atoms in the ligands were scaled by a factor of 0.80, applying a partial atomic charge cutoff of 0.15 to enhance ligand flexibility and optimise interaction profiles. For each ligand, up to ten distinct binding poses were generated. The top-ranking conformations, as determined by Glide docking scores, were selected for further analysis. All docked poses were manually examined to evaluate the presence of key interactions, such as hydrogen bonding, hydrophobic contacts, and π–π stacking, within the defined binding site^27,28^.

### Molecular dynamics simulations

To investigate the structural stability and dynamic behaviour of the inhibitors and Eg5 complex, Desmond, a molecular simulation package, was utilised^29^. The best docking conformations were used as initial structures for the simulation studies. For the system setup, each protein–ligand complex was embedded in an orthorhombic box of TIP3P water molecules, maintaining a buffer distance of 10 Å from the protein surface. The system was neutralised by adding appropriate counterions (Na⁺ or Cl⁻), and a physiological salt concentration of 0.15 M NaCl was added to mimic biological conditions. The OPLS4 force field was used to parameterise all components of the system. Further, before the production run, the system was subjected to energy minimization using a hybrid steepest descent and limited-memory Broyden–Fletcher–Goldfarb–Shanno (L-BFGS) algorithm to relieve steric clashes. A standard Desmond relaxation protocol was employed, consisting of a series of restrained and unrestrained equilibration steps under NVT and NPT ensembles, progressively heating the system to 300 K. Finally, the equilibrated system was subjected to a 100 ns production run under the NPT ensemble, maintaining the temperature at 300 K using the Nose–Hoover thermostat and pressure at 1.01325 bar using the Martyna-Tobias-Klein barostat. The RES-PA integrator was used with a time step of 2 fs, and coordinates were saved every 100 ps for trajectory analysis.

### Principal component analysis (PCA) based free energy landscape (FEL) of protein-ligand complex

PCA was performed on the dynamic simulation trajectory file. Before PCA, the trajectories were pre-processed to remove translational and rotational motions by aligning all frames to the initial reference structure using backbone atoms. PCA was then conducted using the essential dynamics module from Schrodinger. Atom selections were made based on the backbone atoms (N, Cα, C) of the protein to capture dominant conformational motions. The covariance matrix of atomic positional fluctuations was calculated over the aligned trajectory, and eigenvectors and corresponding eigenvalues were obtained through diagonalisation. The first few principal components (typically PC1-PC2), representing the largest conformational variances, were retained for further analysis. Visualisation of motions along principal components was carried out. While 2D projections of conformational sampling along PC1 and PC2 were generated to evaluate clustering and conformational transitions^30^.

### Dynamic cross correlation matrix (DCCM) analysis

For DCCM computation, the trajectory was analysed to extract backbone, Cα atom coordinates across the simulation trajectory. The default parameters were used, stating: time interval = full trajectory, atoms = Cα, and reference structure = initial frame. The cross-correlation coefficient Cij between atomic fluctuations of residues i and j was calculated using the equation, $c_{{ij}} = {{\left\langle {\Delta ri \cdot \Delta rj} \right\rangle } \mathord{\left/ {\vphantom {{\left\langle {\Delta ri \cdot \Delta rj} \right\rangle } {\left( {\left| {\Delta ri} \right|\left| {\Delta rj} \right|} \right)}}} \right. \kern-\nulldelimiterspace} {\left( {\left| {\Delta ri} \right|\left| {\Delta rj} \right|} \right)}}$, where Δri and Δrj represent the displacement vectors of atoms i and j from their mean positions. The resulting matrix values range from + 1 (fully correlated motion) to − 1 (fully anti-correlated motion), with 0 indicating no correlation. The Cross-correlation Matrix was obtained *via* the simulation interaction diagram module and further analysed to identify regions of coordinated dynamics relevant to functional conformational changes^30^.

## Results and discussion

### Identification of bioactives in *A. altissima*

The phytochemical constituents of *A. altissima* were identified through a combination of literature mining from published research articles and data retrieval from HERB 2.0, a high-throughput experiment- and reference-guided database for traditional Chinese medicine. The HERB database entry for *A. altissima* (HERB ID: HERB000838) provided comprehensive information on its reported compounds. A total of 45 compounds (Supplementary File S1) were initially identified, out of which 25 compounds were selected for further analysis based on the availability of corresponding target information and the exclusion of single-chain molecules, ensuring the relevance and suitability of the compounds for downstream bioinformatics and pharmacological studies.

### Acquisition of active targets for the treatment of lung cancer from *A. Altissima*

**Fig. 1:**
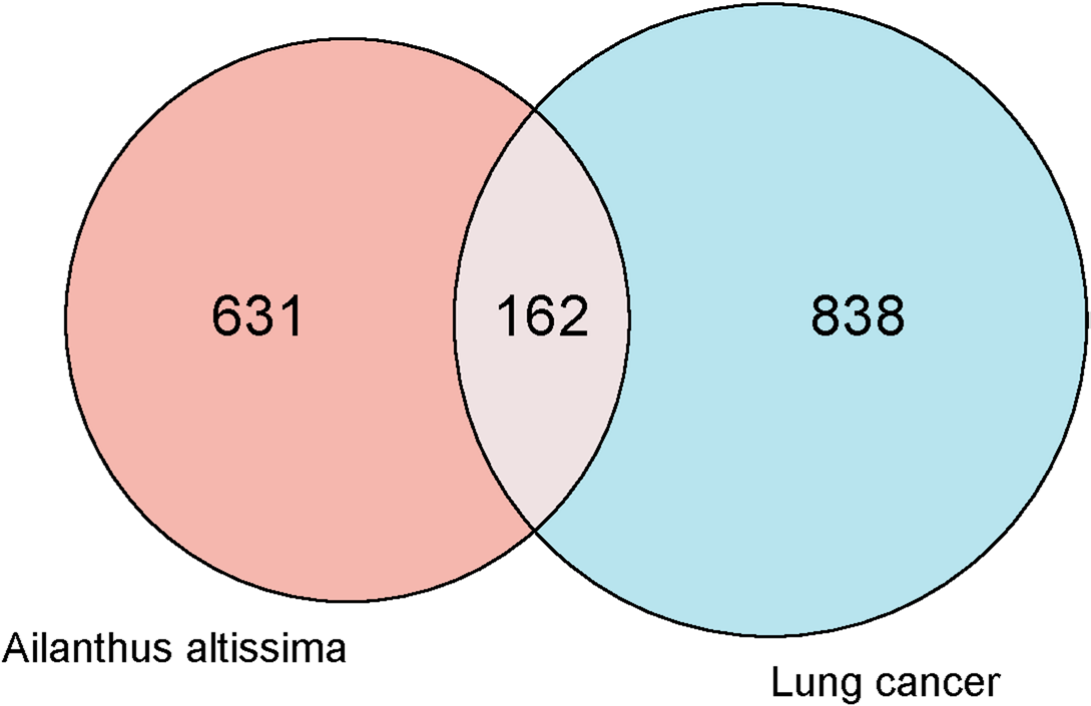
Common targets between *A. altissima* and lung cancer.

Potential targets for the 25 selected compounds from *A. altissima* were predicted using the SwissTargetPrediction database, yielding a total of 631 predicted protein targets. To identify disease-related targets, the GeneCards database was queried using the keyword “lung cancer,” resulting in 838 lung cancer (LC)-related genes based on relevance scores (Supplementary File S2). A comparison of these datasets revealed 162 overlapping genes between *A. altissima* targets and lung cancer-related genes, representing potential therapeutic targets, as depicted in Fig. 1.

### Protein-protein interaction (PPI) network

**Fig. 2:**
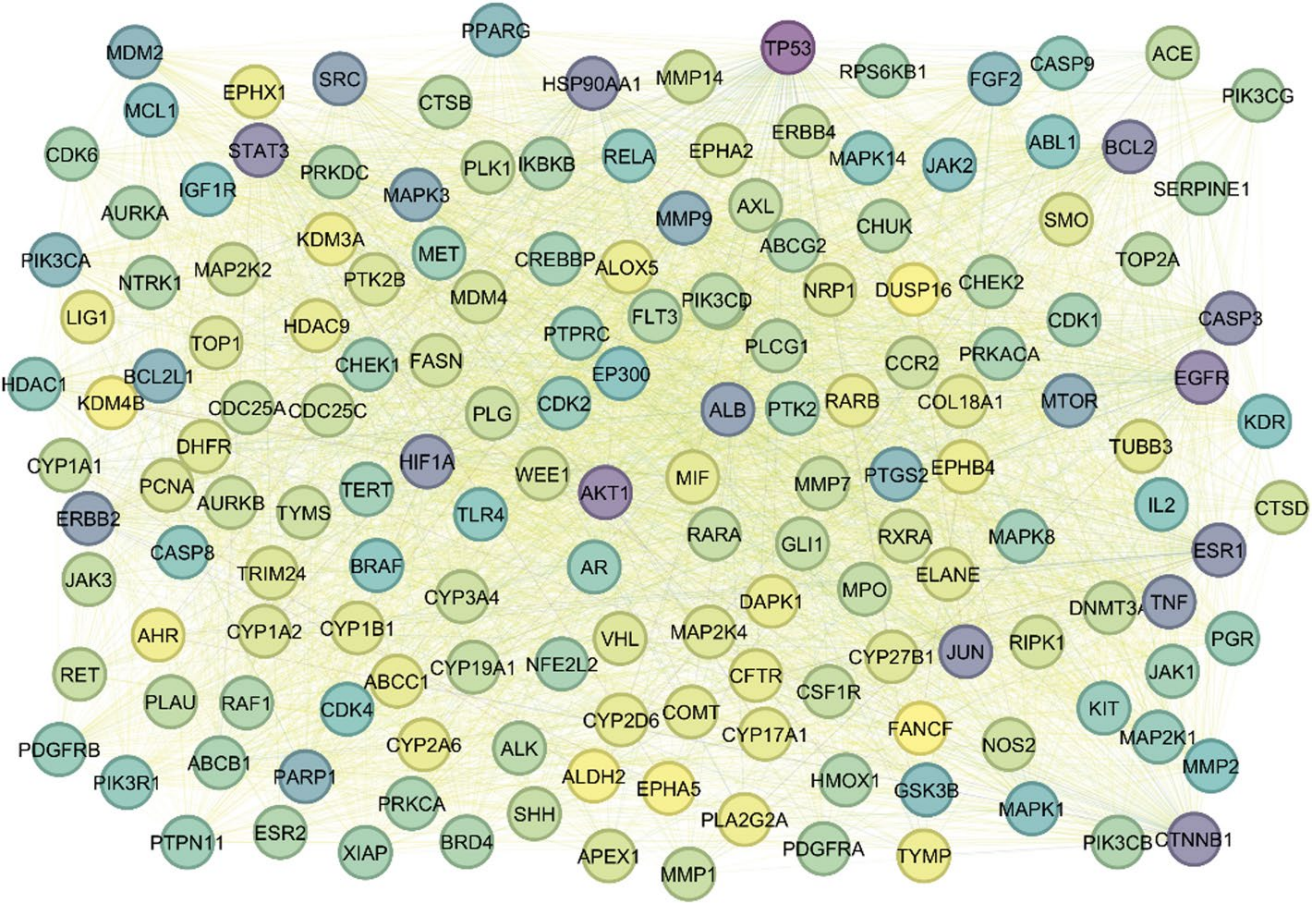
Protein-Protein Interaction (PPI) network.

**Fig. 3:**
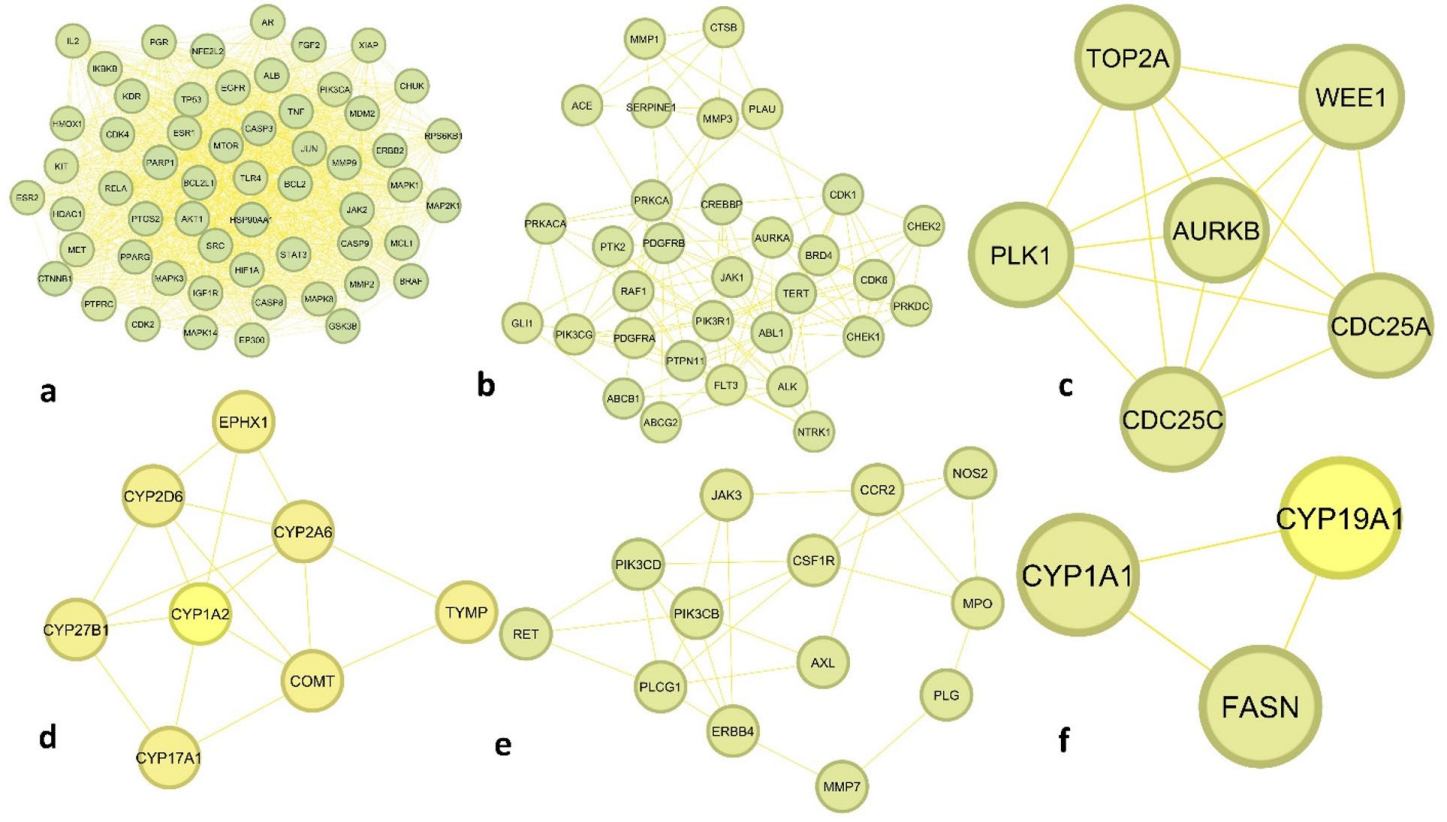
MCODE (Molecular Complex Detection) analysis.

The Protein-Protein Interaction (PPI) network constructed for lung cancer encompasses 162 nodes and 4027 edges, forming a dense and highly interconnected system of molecular interactions critical to tumor development, progression, and therapeutic intervention (Fig. 2). Central hub proteins such as TP53, AKT1, EGFR, CTNNB1, STAT3, BCL2, CASP3, HSP90AA1, JUN, and ESR1 exhibit high degree centrality (ranging from 143 to 113), reflecting their extensive connectivity and regulatory influence, with TP53 standing out for its highest degree (143) and closeness centrality (0.8994), underscoring its key role in tumor suppression, apoptosis, and DNA repair. To further dissect this network, MCODE (Molecular Complex Detection) analysis was employed to identify densely connected modules with potential therapeutic implications. MCODE (Molecular Complex Detection) analysis identified six significant clusters with potential therapeutic relevance (Table 1). Cluster 1, with the highest score (48.25) and 57 nodes, represents a central hub of oncogenic and regulatory proteins including AKT1, TP53, EGFR, MAPKs, MTOR, and STAT3, known to drive lung cancer progression and drug resistance. Cluster 2 (score 8.645; 32 nodes) comprises targets like AURKA, CDK1, TERT, and BRD4, associated with cell cycle dysregulation and genomic instability. Smaller clusters, such as Cluster 3 (score 6; 6 nodes), include mitotic regulators like PLK1 and TOP2A, while Cluster 4 (score 4.857; 8 nodes) features drug-metabolizing enzymes (CYPs, COMT), linked to chemoresistance. Clusters 5 and 6, with scores of 4.667 and 3 respectively, include key signaling and metabolic mediators such as PI3K isoforms, ERBB4, FASN, and CYP19A1, involved in inflammatory signaling, lipid metabolism, and tumor proliferation (Fig. 3). Together, this integrative network and cluster analysis highlights critical molecular hubs and modules, offering a strategic foundation for identifying and prioritizing druggable targets in lung cancer therapy.

**Table 1:** MCODE (Molecular complex Detection) analysis.

Cluster	Score	Nodes	Edges	Targets
1	48.25	57	1351	AKT1, ALB, AR, BCL2, BCL2L1, BRAF, CASP3, CASP8, CASP9, CDK2, CDK4, CHUK, CTNNB1, EGFR, EP300, ERBB2, ESR1, ESR2, FGF2, GSK3B, HDAC1, HIF1A, HMOX1, HSP90AA1, IGF1R, IKBKB, IL2, JAK2, JUN, KDR, KIT, MAP2K1, MAPK1, MAPK14, MAPK3, MAPK8, MCL1, MDM2, MET, MMP2, MMP9, MTOR, NFE2L2, PARP1, PGR, PIK3CA, PPARG, PTGS2, PTPRC, RELA, RPS6KB1, SRC, STAT3, TLR4, TNF, TP53, XIAP
2	8.645	32	134	ABCB1, ABCG2, ABL1, ACE, ALK, AURKA, BRD4, CDK1, CDK6, CHEK1, CHEK2, CREBBP, CTSB, FLT3, GLI1, JAK1, MMP1, MMP3, NTRK1, PDGFRA, PDGFRB, PIK3CG, PIK3R1, PLAU, PRKACA, PRKCA, PRKDC, PTK2, PTPN11, RAF1, SERPINE1, TERT
3	6	6	15	AURKB, CDC25C, CDC25A, PLK1, TOP2A, WEE1
4	4.857	8	17	EPHX1, CYP2D6, CYP27B1, CYP1A2, COMT, CYP17A1, TYMP, CYP2A6
5	4.667	13	28	NOS2, CCR2, CSF1R, JAK3, PIK3CD, PIK3CB, AXL, ERBB4, PLCG1, RET, MMP7, PLG, MPO
6	3	3	3	FASN, CUP1A1, CYP19A1

### Gene ontology (GO) enrichment of analysis

**Fig. 4:**
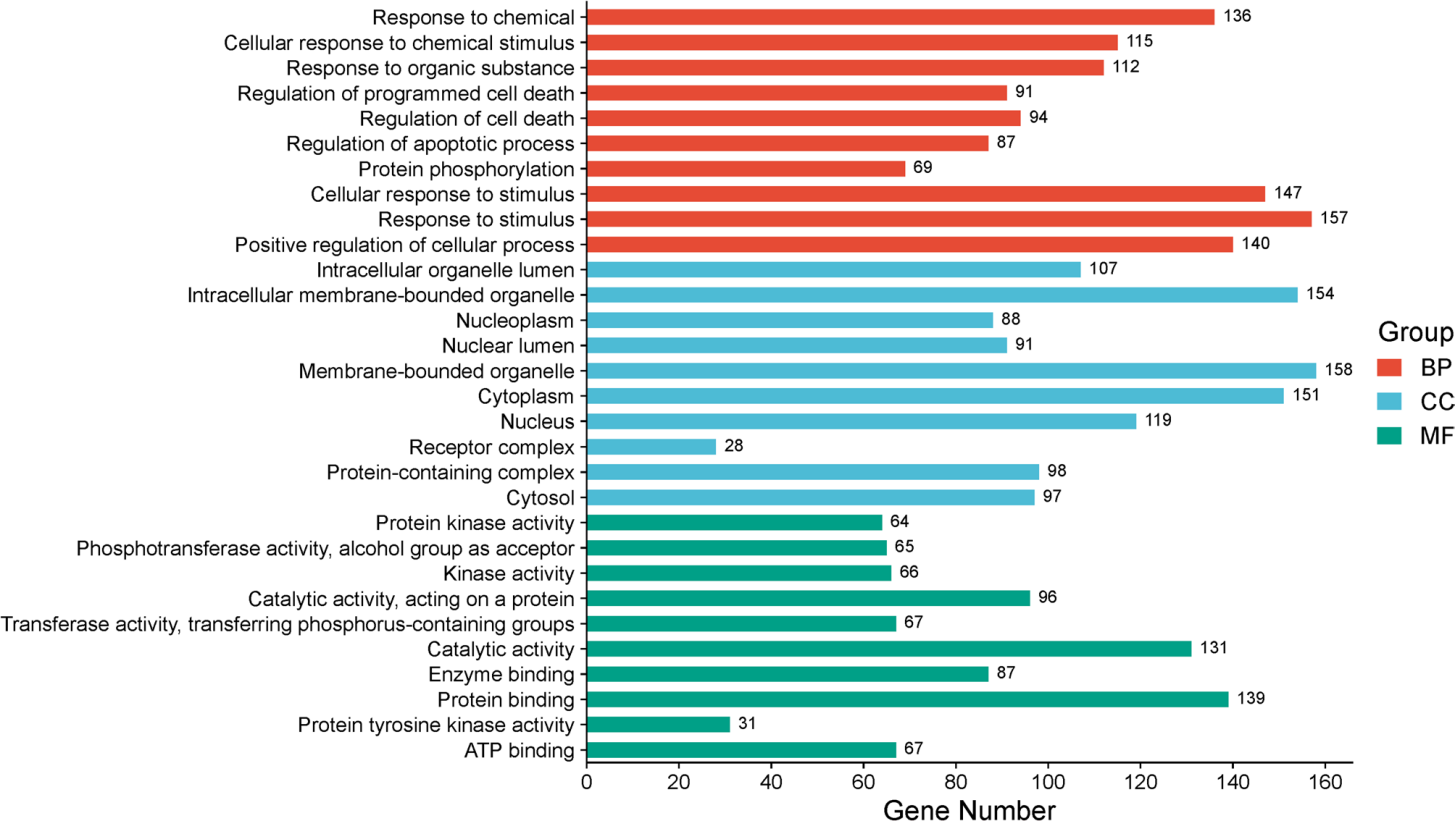
Gene ontology enrichment of analysis of *A. altissima* for treatment of lung cancer.

Gene Ontology (GO) enrichment analysis of lung cancer-related targets reveals a comprehensive network of biological processes, cellular components, and molecular functions central to disease pathophysiology and therapeutic intervention (Supplementary File S3). Biologically, key processes such as response to chemical stimuli (GO:0042221, GO:0070887) and regulation of apoptosis and cell death (GO:0043067, GO:0010941, GO:0042981) involve critical genes like EGFR, MAPKs, AKT1, TP53, BCL2, CASP3/8/9, and MCL1, which mediate cellular adaptation to drugs, restore apoptosis, and modulate chemoresistance. Protein phosphorylation (GO:0006468) and positive regulation of cellular processes (GO:0048522) highlight survival signaling pathways involving PIK3CA, BRAF, STAT3, and others, often dysregulated in lung cancer. The cellular component analysis localizes these targets to strategic intracellular compartments such as the cytoplasm (GO:0005737) and cytosol (GO:0005829), where kinases like MAPK1, AKT1, and JAK2 operate; the nucleoplasm (GO:0005654) and nuclear lumen (GO:0031981), which house transcriptional regulators and tumor suppressors like TP53, EP300, and HIF1A; and membrane-bound organelles (GO:0043227) and receptor complexes (GO:0043235), including EGFR, ERBB2, MET, and FLT3, that initiate oncogenic signaling and serve as key targets of tyrosine kinase inhibitors (TKIs). At the molecular function level, enriched terms such as protein kinase activity (GO:0004672) and protein tyrosine kinase activity (GO:0004713) encompass a wide array of oncogenic kinases (EGFR, BRAF, JAKs, PI3KCA), while ATP binding (GO:0005524) and enzyme binding (GO:0019899) reflect the enzymatic dependence of these pathways, targeted by ATP-competitive inhibitors and enzyme antagonists such as PARP inhibitors and MDM2 blockers (Fig. 4). These GO insights provide an integrated molecular and spatial framework of lung cancer pathology and therapy, identifying critical targets across signaling, transcriptional regulation, and apoptosis that can be leveraged to enhance precision treatment and overcome resistance.

### Gene ontology-immuno system process

**Fig. 5:**
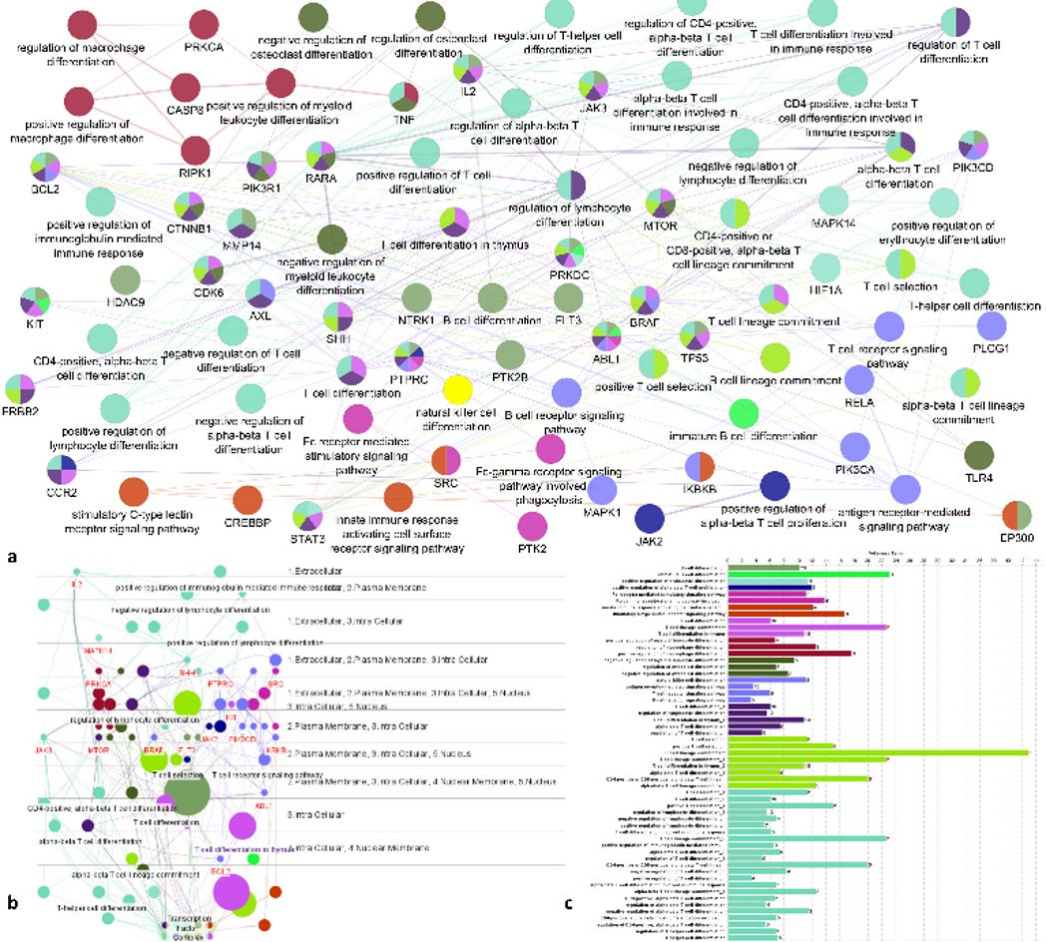
(**a**) ClueGo representation of immune system process. (**b**): Extracellular and intracellular processes of immune system process (**c**): Bar graph representation of immune system process.

Gene Ontology (GO) terms of immune system processes identified by using Clue Go and Cluepedia plugins that are intricately involved in lung cancer pathogenesis and immune regulation (Fig. 5). Central to these are processes involving T cell differentiation (GO:0030217), T cell lineage commitment, and regulation of T-helper and CD4+/CD8 + T cell pathways, reflecting the critical role of adaptive immunity in tumor recognition and immune escape. Genes like IL2, JAK3, RARA, STAT3, and SHH appear repeatedly, suggesting they serve as key regulatory nodes in modulating T cell and B cell differentiation, activation, and signaling. Notably, the involvement of genes such as PIK3R1, TP53, and BCL2 also implicates cross-talk between oncogenic pathways and immune functions. Additionally, components such as Fc receptor signaling (GO:0002431) and natural killer (NK) cell differentiation underscore the role of innate immunity. The recurrence of both positive and negative regulatory processes (regulation of lymphocyte and macrophage differentiation) reveals a finely balanced immune landscape in lung cancer, where tumors manipulate immune processes to promote immune evasion, inflammation, or tolerance. Overall, these GO terms and their associated genes demonstrate the complexity of immune modulation in lung cancer and highlight potential targets for immunotherapeutic intervention.

### KEGG pathway analysis

**Fig. 6:**
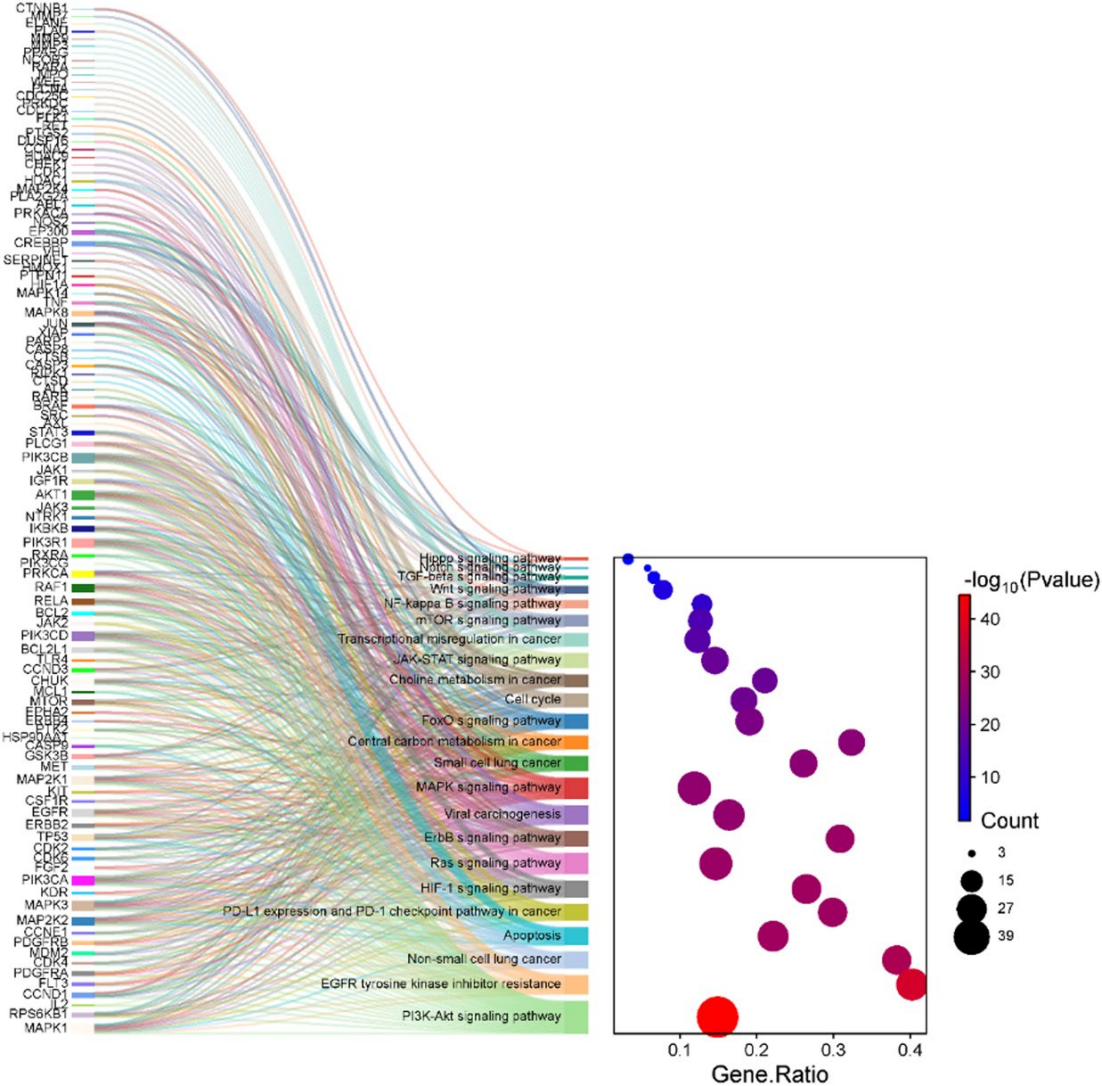
KEGG pathway analysis of selected signaling pathways involved in the treatment of lung cancer.

The KEGG pathway analysis reveals multiple mechanisms involved in lung cancer pathogenesis and treatment, with key contributions from pathways such as non-small cell lung cancer (hsa05223), PI3K-Akt signaling (hsa04151), EGFR tyrosine kinase inhibitor resistance (hsa01521), MAPK signaling (hsa04010), apoptosis (hsa04210), and PD-L1/PD-1 checkpoint pathway (hsa05235). Central genes implicated across these include MAPK1, MAPK3, MAP2K1/2, PIK3CA, EGFR, ERBB2, TP53, CDK4/6, STAT3, AKT1, and BCL2 family members. The non-small cell lung cancer pathway (hsa05223) integrates aberrant EGFR signaling, frequently mutated in lung tumors, leading to activation of the PI3K-Akt and MAPK cascades, promoting cell proliferation, survival, and resistance to apoptosis. The PI3K-Akt pathway (hsa04151) mediates survival and growth by phosphorylating downstream effectors such as mTOR, BCL2, and FOXO, while the MAPK pathway (hsa04010), activated via RTKs like EGFR and PDGFRA, regulates gene expression and mitosis. EGFR TKI resistance (hsa01521) is driven by compensatory activation of MET, AXL, and BRAF, which bypass EGFR blockade and maintain downstream signaling. Apoptosis dysregulation (hsa04210) *via* inhibition of CASP3, CASP9, and overexpression of BCL2/BCL2L1 enables tumor cells to evade death. Additionally, immune evasion is promoted by PD-L1 expression and PD-1 checkpoint pathway (hsa05235), where genes like STAT3, AKT1, and HIF1A upregulate PD-L1, allowing tumor cells to suppress T-cell activity. These interconnected pathways present therapeutic targets for kinase inhibitors (EGFR, ALK, BRAF), immune checkpoint inhibitors (anti-PD1/PDL1), and pro-apoptotic agents, forming the molecular basis for targeted and immunotherapy approaches in lung cancer treatment (Fig. 6).

### Network construction between bioactives, shared key targets, and signalling pathways

**Fig. 7:**
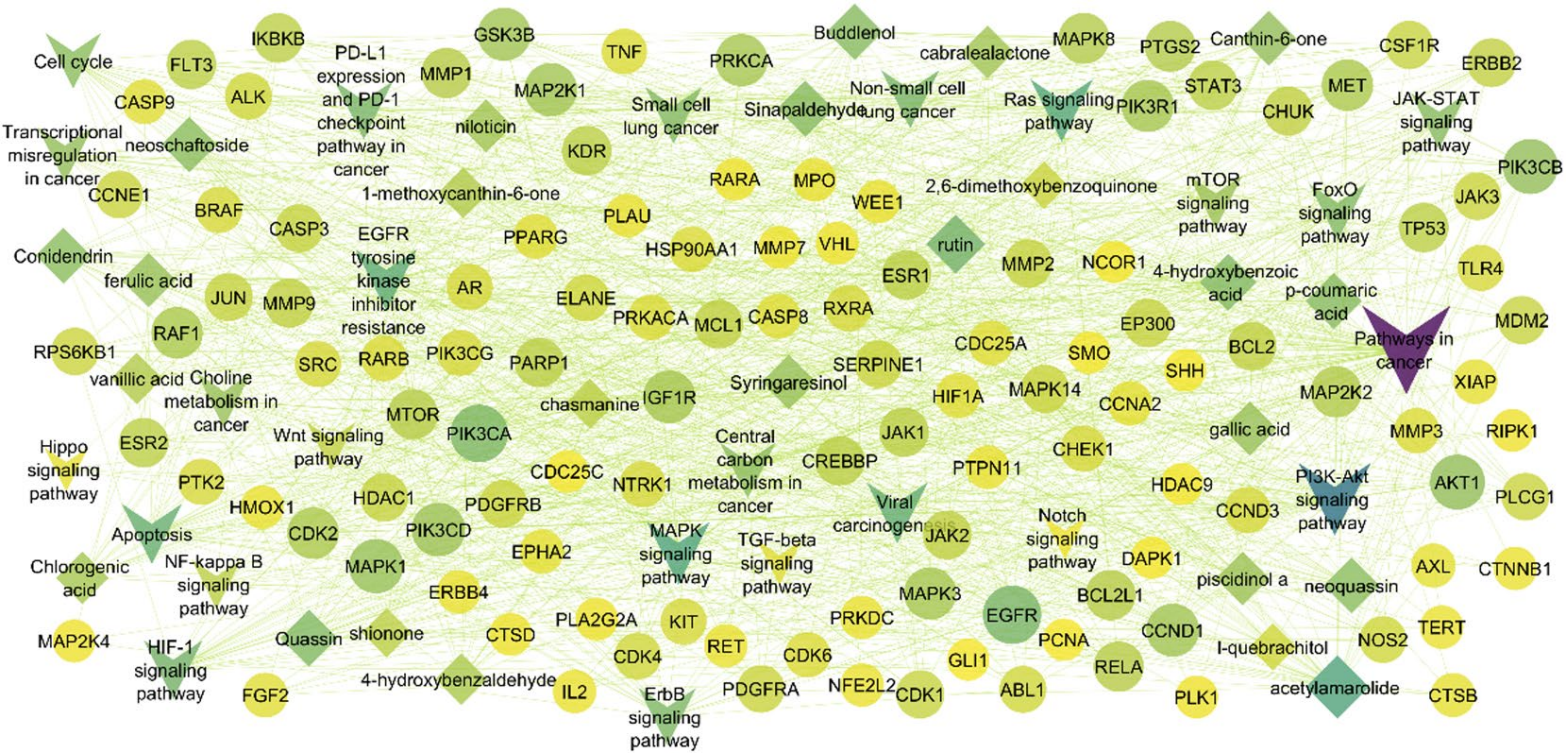
Network construction between compounds, targets, and signaling pathways.

To systematically elucidate the underlying molecular mechanisms of *A. altissima* in the treatment of lung cancer, a comprehensive compound-target-pathway network was constructed using Cytoscape 3.10.3 (Fig. 7). This integrative network, comprised of 166 nodes and 1,098 edges, encapsulating the complex interactions among bioactive compounds, predicted targets, and enriched signaling pathways. In this network, diamond-shaped nodes represent active compounds, ellipses denote target genes, and V-shaped nodes indicate significantly enriched pathways. The network topology reveals a multi-target, multi-pathway pharmacological profile, where each compound potentially modulates several targets, and individual targets are implicated in multiple signaling pathways. Notably, the PI3K-Akt signaling pathway and EGFR tyrosine kinase inhibitor resistance pathway emerged as key nodes, interacting with 52 and 34 targets, respectively, suggesting their pivotal roles in the anti-lung cancer activity of *Ailanthus altissima*. Among the 25 compounds analyzed, acetylamarolide exhibited the highest degree of connectivity, targeting 35 key proteins, including CCNE1, CDK2, SRC, MMP9, CASP3, HDAC1, PIK3 family members, and MAPK signaling molecules. Furthermore, EGFR surfaced as a central hub target, being modulated by 13 distinct compounds and linked to 12 critical signaling pathways, highlighting its potential as a key therapeutic target within this botanical network framework. This systems-level analysis provides valuable insights into the polypharmacological mechanisms of *A. altissima* and its therapeutic potential in lung cancer intervention.

### Differential expression of hub genes in lung adenocarcinoma (LUAD) and lung squamous cell carcinoma (LUSC) in the GEPIA database

**Fig. 8:**
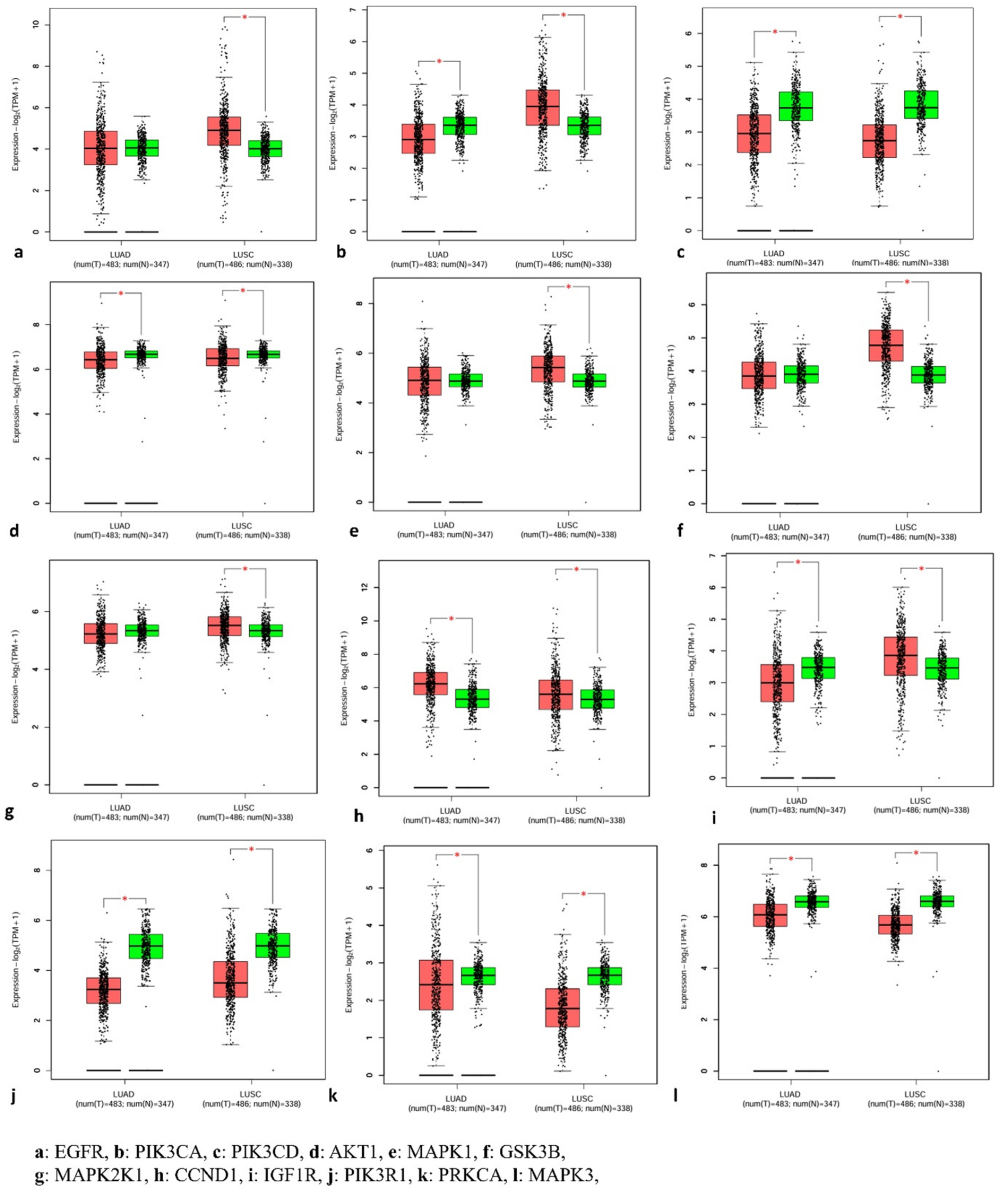
Gene expression of the hub genes in patients with Lung adenocarcinoma (LUAD) and Lung squamous cell carcinoma (LUSC) versus normal controls.

To validate the expression patterns of the identified hub genes in lung cancer, data from TCGA (http://portal.gdc.cancer.gov/) and GTEx were analyzed using the GEPIA2 platform. The results are presented as box plots (Fig. 8), comparing the expression levels of the top 10 hub genes in Lung Adenocarcinoma (LUAD) and Lung Squamous Cell Carcinoma (LUSC) samples against normal lung tissues. The analysis included 483 LUAD tumor samples and 347 normal samples, as well as 486 LUSC tumor samples and 338 normal samples. The box plots for overexpressed hub genes demonstrate significantly higher median expression levels in tumor tissues, indicating their upregulation and possible involvement in cancer progression. In contrast, the downregulated hub genes show lower expression levels in tumor tissues compared to normal tissues, suggesting a potential tumor suppressor role. Overall, the differential expression patterns of these hub genes imply their contribution to lung cancer pathogenesis through abnormal gene regulation.

### Prognostic relevance of hub genes in the GEPIA database for lung adenocarcinoma (LUAD) and lung squamous cell carcinoma (LUSC)

**Fig. 9:**
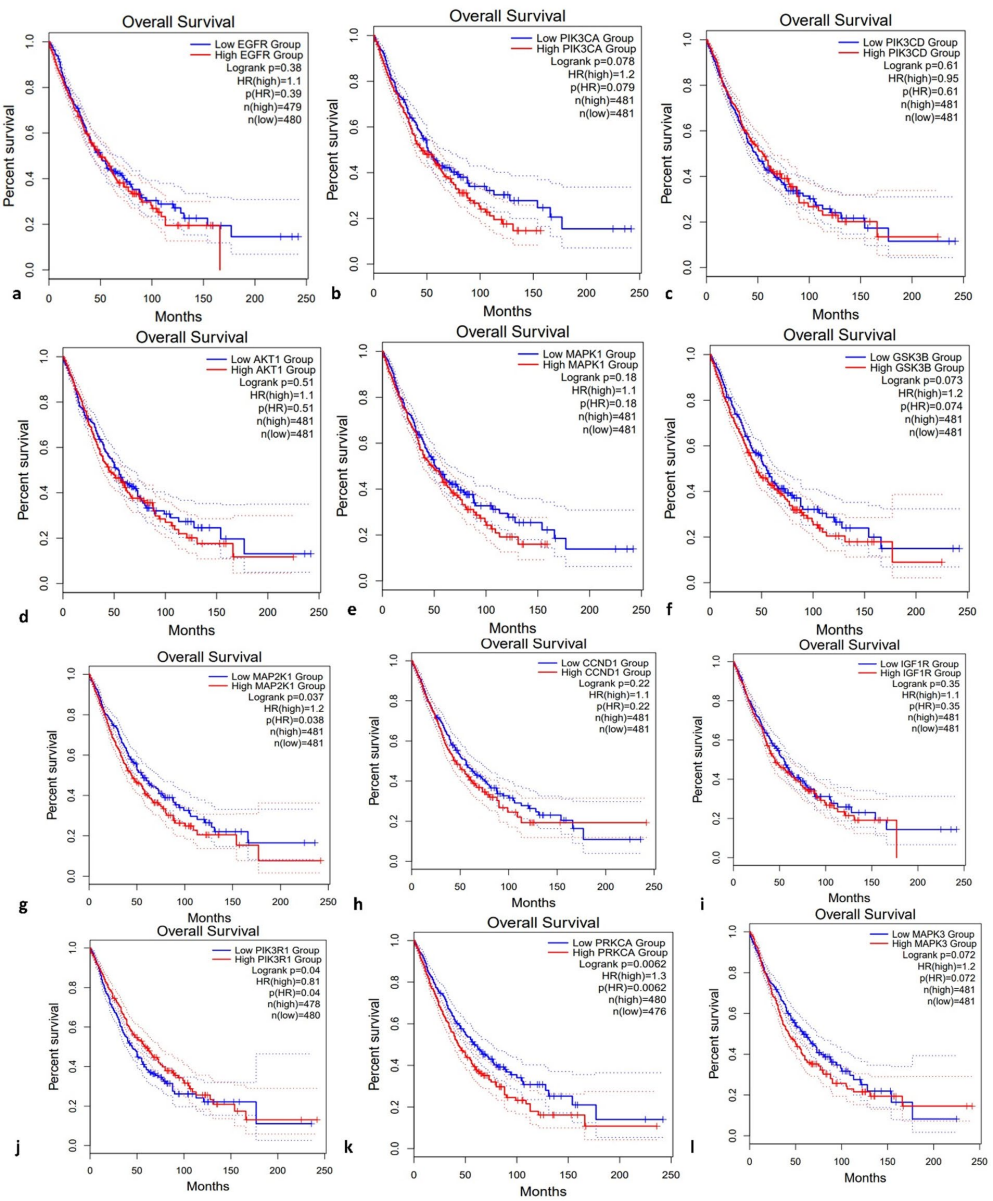
Survival curves of hub genes in Lung adenocarcinoma (LUAD) and Lung squamous cell carcinoma (LUSC) from the GEPIA database. (**a**) EGFR, (**b**) PIK3CA, (**c**) PIK3CD, (**d**) AKT1, (**e**) MAPK1, (**f**) GSK3B, (**g**) MAP2K1, (**h**) CCND1, (**i**) IGF1R, (**j**) PIK3R1, (**k**) PRKCA, (**l**) MAPK3. Kaplan Meier survival curves were generated, stratifying patients into high- and low-expression groups on the basis of a 50% TPM threshold.

The survival analysis illustrated in the figure shows Kaplan-Meier overall survival (OS) curves for patients with Lung Adenocarcinoma (LUAD) and Lung Squamous Cell Carcinoma (LUSC) based on the expression levels of 12 hub genes: EGFR, PIK3CA, PIK3CB, AKT1, MAPK1, GSK3B, MAP2K1, CCND1, IGF1R, PIK3R1, PRKCA, and MAP3K9 (Fig. 9). In general, higher expression of several hub genes, including EGFR, PIK3CA, PIK3CB, AKT1, MAPK1, GSK3B, PIK3R1, and PRKCA, is associated with poorer survival outcomes, as evidenced by the red curves being consistently lower than the blue ones (low-expression groups), indicating reduced survival probability over time. For example, EGFR shows a noticeable separation between the high and low expression groups, with a hazard ratio (HR) of 1.31, suggesting a 31% higher risk of death in patients with high EGFR expression. Similarly, PIK3CB (panel c) and PRKCA (panel k) show significant prognostic value with log-rank *p* values < 0.05 and HRs of 1.5 and 1.34, respectively.

Conversely, genes such as MAP2K1, CCND1, and IGF1R show less pronounced differences between high and low expression groups, with non-significant p-values indicating limited prognostic relevance in LUAD/LUSC. Some genes, like MAPK3 and MAPK1, show an HR of 1.1 but with *p* values > 0.05, suggesting a trend toward worse survival with higher expression but not statistically significant. Overall, the survival analysis suggests that upregulation of genes involved in PI3K-Akt and MAPK signaling pathways correlates with poorer outcomes in LUAD and LUSC patients. These hub genes may serve as potential prognostic biomarkers and therapeutic targets in lung cancer, particularly for subtypes with high oncogenic pathway activation.

### Molecular docking

**Fig. 10:**
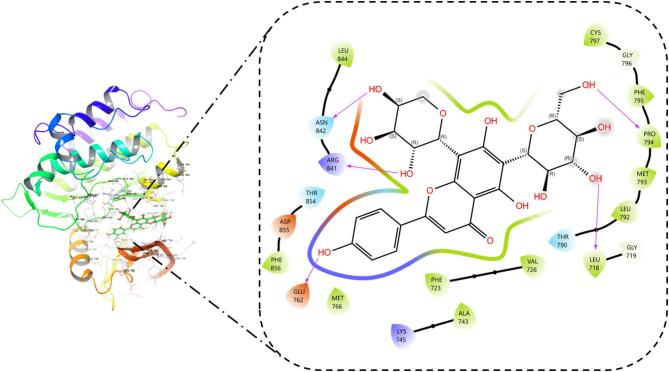
Binding orientation of neoschaftoside in the ligand binding domain of EGFR.

**Fig. 11:**
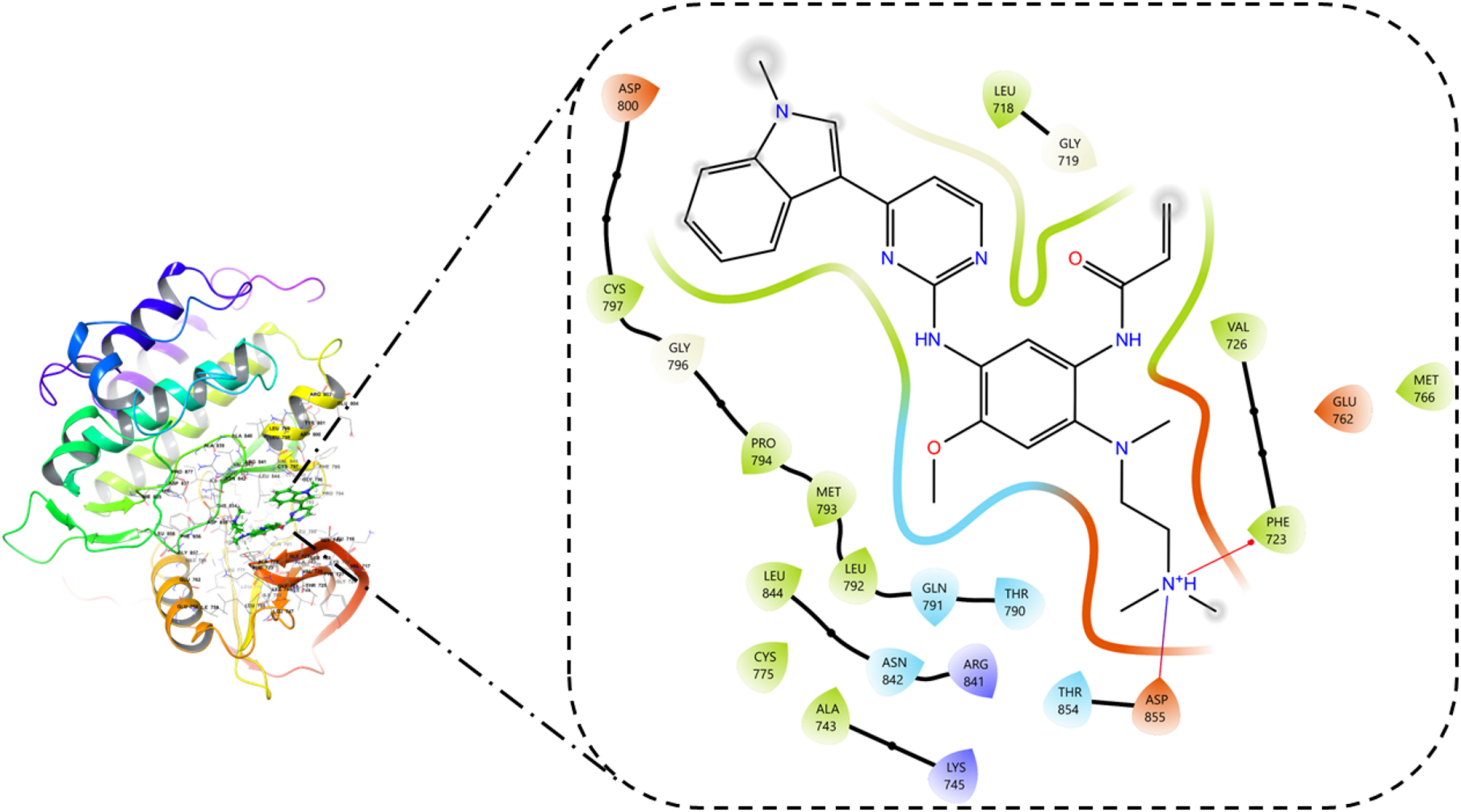
Binding orientation of Osimertinib in the ligand binding domain of EGFR.

**Fig. 12:**
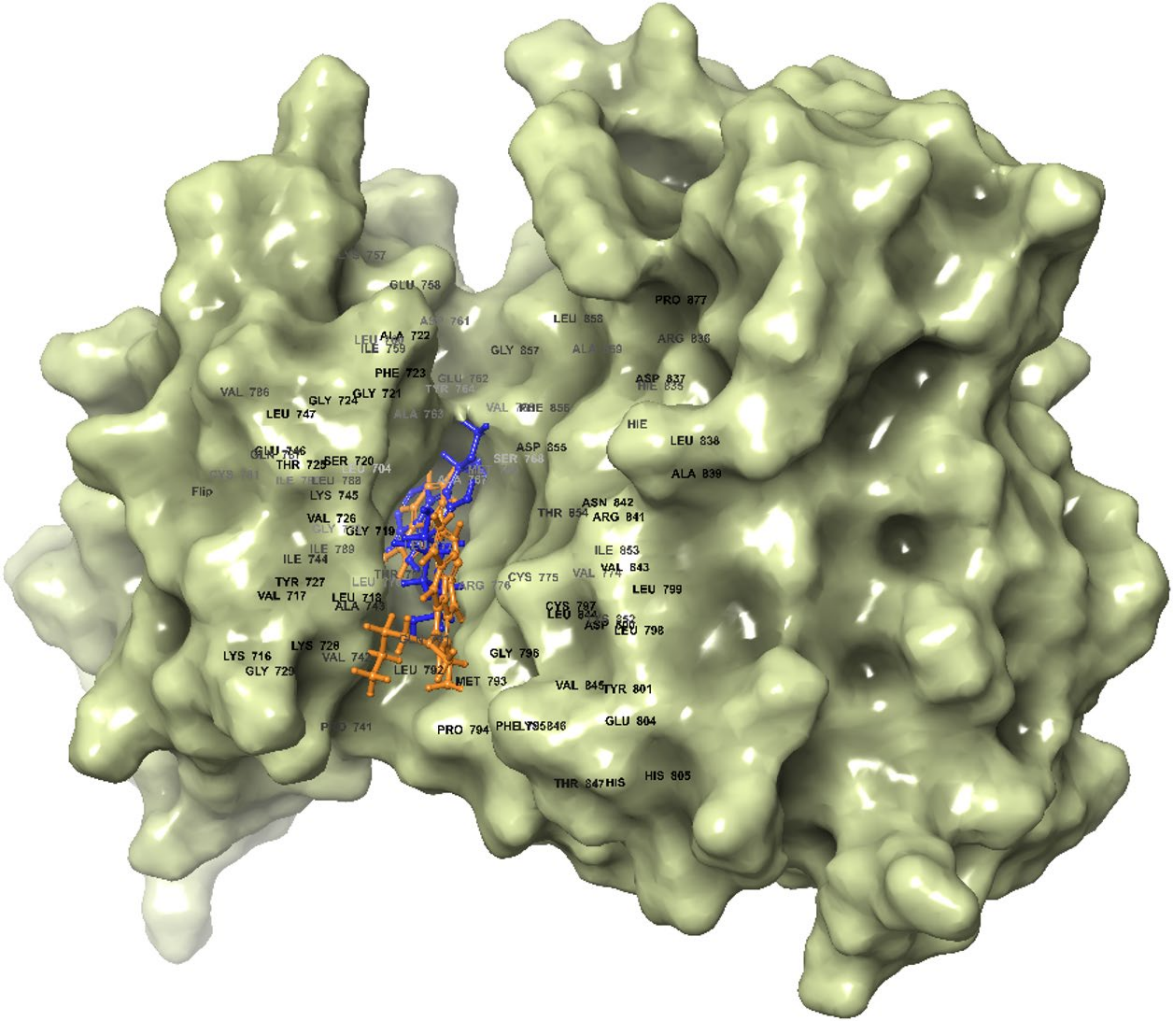
Common binding mode of neoschaftoside (Blue) and Osimertinib (orange) in the ligand binding domain of EGFR.

**Fig. 13:**
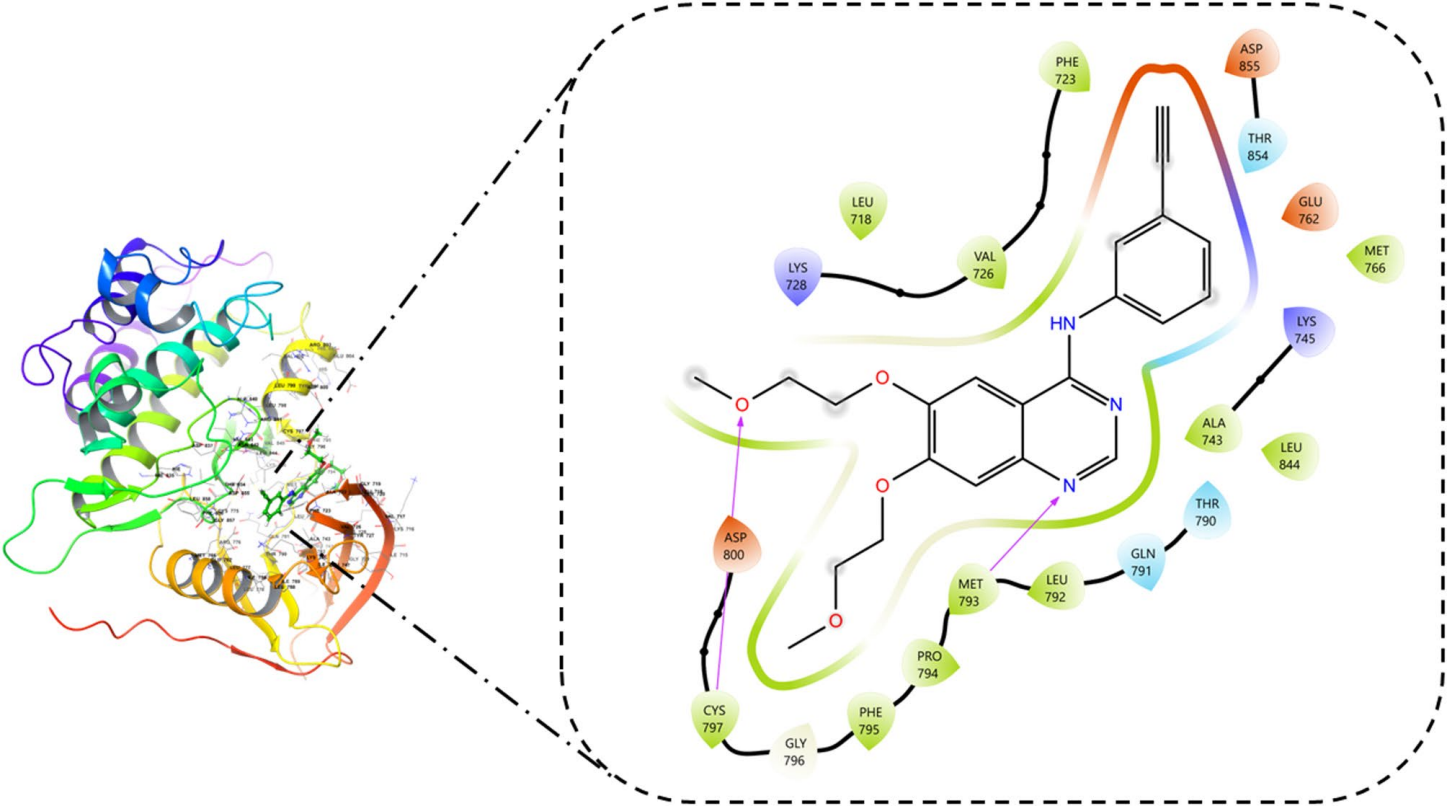
Binding orientation of Erlotinib in the ligand binding domain of EGFR.

Molecular docking was performed to predict the hypothetical binding mode of the bioactives from *Ailanthus altissima*. All the compounds displayed non-bonding interactions. Among all the bioactives from *Ailanthus altissima*, neoschaftoside displayed the better docking score (− 10.765) owing to its energetics (− 63.293 kcal/mol) and binding affinity. It formed hydrogen bond interaction with PRO 794, THR 718, GLU 762, ARG 84, and ASN 842 amino acid residues. Moreover, it formed hydrophobic contacts with MET 793, LEU 792, PRO 794, and VAL 726, as shown in the Fig. 10. Similarly, the Docking methodology was validated by redocking the cocrystal inhibitor (Osimertinib) present in the ligand binding domain of EGFR (4ZAU) protein, which displayed a docking score (− 7.302) and binding energy contribution of − 53.397 kcal/mol, it formed hydrophobic contacts with LEU 844, VAL 845, CYS 797, PRO 794, MRT 793, LEU 792, ILE 789, and LEU 788 as shown in Fig. 11. The RMS De*via*tion for the neoschaftoside and Osimertinib sampled in the binding domain of EGFR was found to be 1.32Å as shown in Fig. 12. Similarly, Erlotinib was considered as the standard EGFR inhibitor, which displayed a docking score of (− 7.925) and energy contributions of − 47.687 kcal/mol. It showed hydrogen bonding with residues like CYS 797 and MET 793. Moreover, it showed hydrophobic contacts with amino acid residues like LEU 792, ALA 743, VAL 726, PHE 795, and PRO 794 as shown in Fig. 13. Likewise, in comparison to cocrystal inhibitor (Osimertinib), seven bioactives showed better binding affinity as summarized in Table 2.

**Table 2:** Binding affinity and interaction results of bioactives in the ligand binding domain of EGFR (4ZAU) protein.

Compound	Docking score	Glide energy (kcal/mol)	Ligand atom interaction (H-Bond)/ π-cation	Bond length
Neoschaftoside	− 10.765	− 63.293	–OH of ligand to PRO794 –OH of ligand to LEU718 –OH of ligand to ASN842 –OH of ligand to ARG841 –OH of ligand to GLU762	2.43 1.87 1.83 1.71 1.68
Rutin	− 10.525	− 58.6	–OH of ligand to ARG 841 –OH of ligand to ASN 842 –OH of ligand to ASP855 –OH of ligand to LEU718	2.09 2.16 2.17 1.85
Buddlenol	− 7.345	− 51.637	–OH of ligand to MET 793 –OH of ligand to GLN 791 –OH of ligand to GLU 804	2.43 1.88 2.01
l-quebrachitol	− 6.804	− 46.193	–OH of ligand to GLU 804 –OH of ligand to GLU 804	1.91 2.12
Erlotinib	− 7.925	− 47.922	–N of ligand to MET 793 –O of ligand to CYS 797	2.13 1.67
Osimertinib	− 7.302	− 53.397	NH of ligand to PHE 723 NH of ligand to ASP 855	2.51 2.81

### Molecular dynamics simulation

**Fig. 14:**
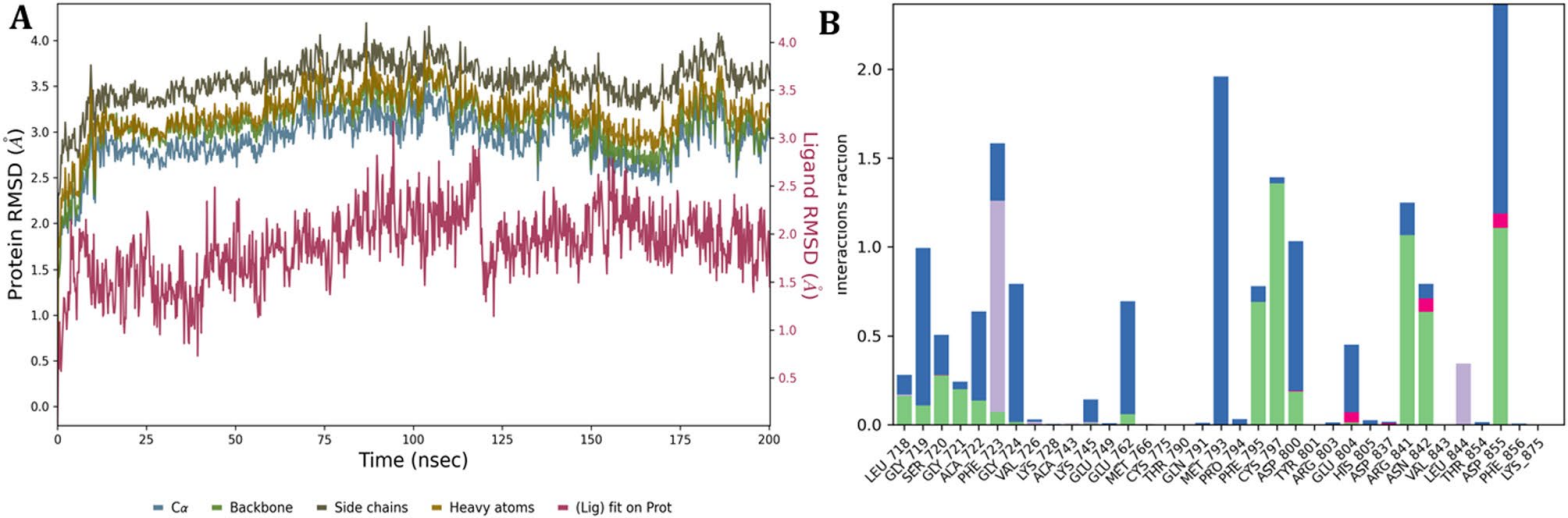
EGFR-Neoschaftoside complex simulation throughout 200 ns, (**A**) Protein-ligand RMSD plot, (**B**) Protein-ligand contact histogram representing the interaction fraction with the amino acids.

The Root Mean Square Deviation (RMSD) profiles of the EGFR-neoschaftoside complex were assessed over a 200 ns MD simulation to evaluate the structural stability and conformational behavior of the protein-ligand complex. The analysis encompassed backbone atoms (Cα, backbone, side chains, and heavy atoms), as well as ligand RMSD, aligned to the protein frame of reference. The Cα RMSD plateaued around an average of 2.9 ± 0.2 Å, indicating that the protein maintained global structural stability with minimal backbone de*via*tion after the initial equilibration phase. Similarly, the backbone RMSD remained consistent throughout the simulation, averaging 3.0 ± 0.2 Å, further supporting the preservation of secondary structural elements. The side chain RMSD showed relatively higher fluctuations, averaging around 3.3 ± 0.3 Å, which is typical due to the inherent flexibility of surface-exposed residues. The heavy atom RMSD of the protein converged to around 3.4 ± 0.2 Å, reflecting the overall compactness and dynamic fluctuations of the protein structure during the simulation period of particular interest. In contrast, the ligand RMSD (fit on protein) remained consistently stable throughout the trajectory, with an average of 1.85 ± 0.4 Å. Although minor transient spikes were observed, there was no evidence of ligand dissociation or major positional drift, suggesting a strong and persistent interaction between the ligand and its binding site. This stable RMSD behavior highlights the favorable binding affinity and conformational restraint imposed by the protein environment on the ligand. The overlay of RMSD curves (Fig. 14A) shows that while the protein components exhibit typical dynamic flexibility, the ligand remains firmly accommodated within the active site. These results collectively underscore the structural integrity of the protein ligand complex and validate the reliability of the predicted binding mode under simulated conditions. To further elucidate the stability of the EGFR- neoschaftoside complex, a contact histogram was generated to assess the nature of residual interactions throughout the 200 ns. The interaction profile revealed that several amino acid residues within the binding pocket exhibited high interaction fractions, suggesting strong and consistent contacts with the ligand. Notably, residues such as VAL724, THR790, GLU804, ARG841, ASP837, and LYS875 displayed interaction fractions close to or exceeding 1.0, indicating that these residues were engaged in ligand binding for a significant portion of the simulation. The high interaction fraction observed for LYS875, in particular, highlights its crucial role in anchoring the ligand, potentially through hydrogen bonding and electrostatic interactions. The different color segments in the histogram correspond to different interaction types, including hydrogen bonds (green), hydrophobic contacts (blue), water bridges (light purple), and π-stacking or other electrostatic interactions (pink). The presence of multiple interaction types with residues like GLU804 and ARG841 suggests a multi binding mechanism, which can enhance the affinity and specificity of the ligand toward the binding site (Fig. 14B). These persistent interactions correlate strongly with the ligand RMSD profile, which remained consistently below 2.5 Å throughout the simulation. The restricted positional drift and lack of substantial changes in the ligand trajectory confirm the importance of these stable connections in preserving the conformational integrity of the complex. Moreover, the stability of protein backbone and sidechain RMSD (averaging between 2.9 and 3.4 Å) complements the interaction data, indicating that the ligand binding did not induce large scale conformational perturbations in the protein structure. This indicated the notion of a well adapted binding pocket, structurally organized to accommodate the ligand with minimal energetic penalties. Thus, the residue interaction analysis supports the RMSD findings by confirming that a network of stable and specific contacts, particularly involving polar and charged residues, contributes to the overall structural stability and sustained binding of the ligand. Together, these insights validate the robustness of the EGFR-neoschaftoside complex.

### Dynamic cross correlation matrix of neoschaftoside bound EGFR protein

**Fig. 15:**
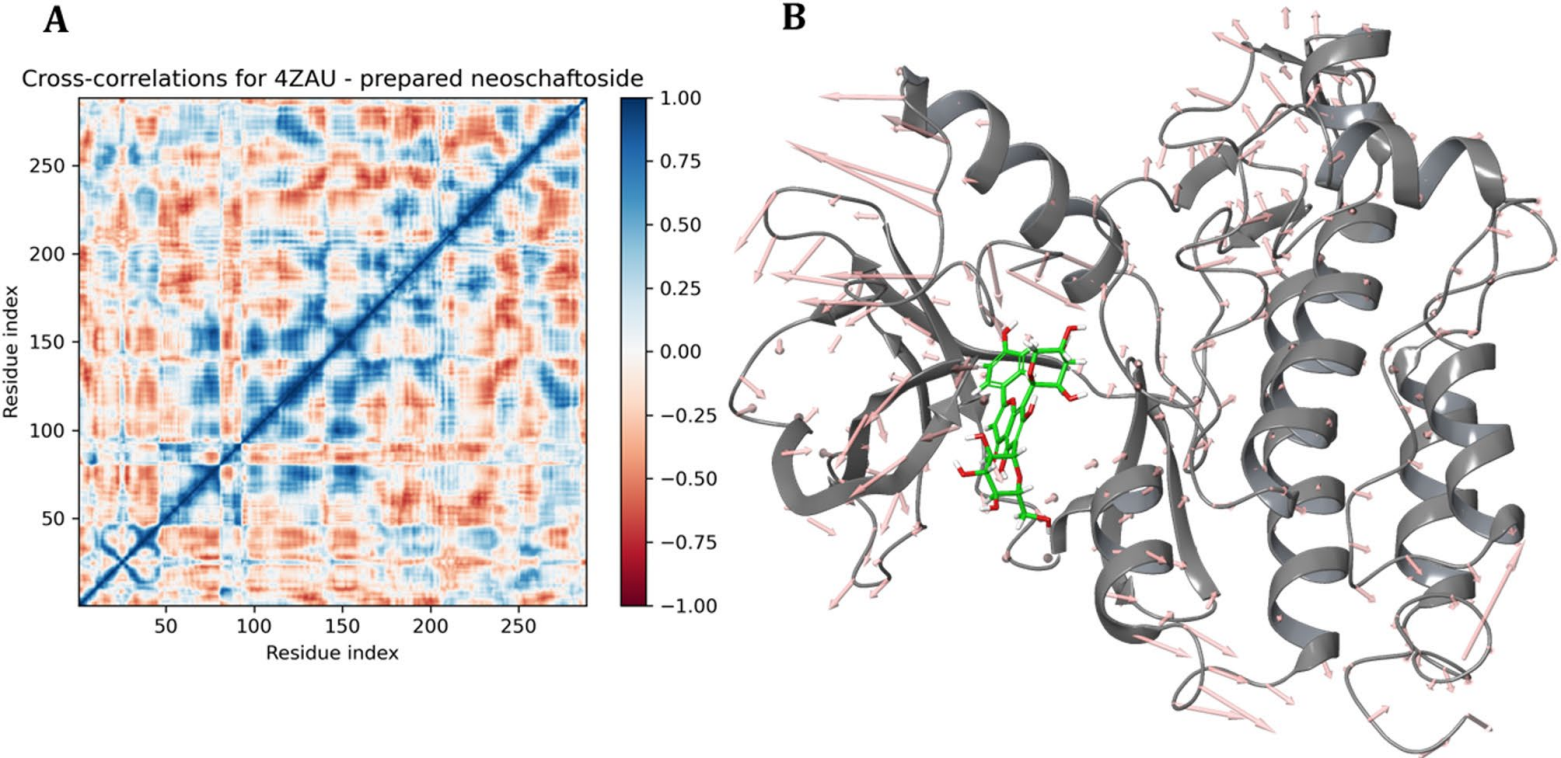
(**A**) An intricate dynamic cross-correlation matrix represented by blue color for positive correlation and red color for negative correlation, (**B**) Arrows representing the eigenvectors for the Cα atoms.

To gain further insight into the dynamic behavior of the EGFR protein in the presence of neoschaftoside, DCCM was computed. Which provides a residual overview of concerted and opposing motions during the 200 ns MD simulation, revealing the extent of correlated fluctuations between atomic pairs across the protein structure. In the DCCM heatmap (Fig. 15), positive correlations (blue) represent residues that move in a concerted direction, while negative correlations (red) indicate anti-correlated movements. Regions near the diagonal display strong self-correlations, while off-diagonal zones highlight domain-level motions and long-range interactions. The analysis revealed prominent regions of positive correlation, particularly among residues spanning the N-lobe and hinge region (residues 40–120), suggesting stable intra-domain cooperative motions in response to ligand binding. These correlated motions likely support the structural integrity and functional conformation of the EGFR kinase domain. In contrast, anti-correlated movements were observed between residues in the C-lobe (residues 150–250) and portions of the N-lobe, suggesting flexible adaptations involved in allosteric modulation. The presence of both correlated and anti-correlated dynamic networks suggests that neoschaftoside binding induces long-range conformational coupling, which could contribute to altered enzymatic activity or stabilization of a specific inactive conformation. The observed correlations are consistent with the relatively low RMSD fluctuations for the ligand, as previously described in the RMSD analysis, thus reinforcing the notion that the ligand maintains a well-adapted fit without disrupting global folding.

### Principal component analysis to predict the free energy landscape

To characterize the conformational space explored by the EGFR-neoschaftoside complex, PCA was performed, and the corresponding FEL was constructed based on the first two principal components (PC1 and PC2). The PCA technique reduces the dimensional complexity of molecular motions and highlights collective, large-scale movements that contribute most to the structural variance during the simulation.

**Fig. 16:**
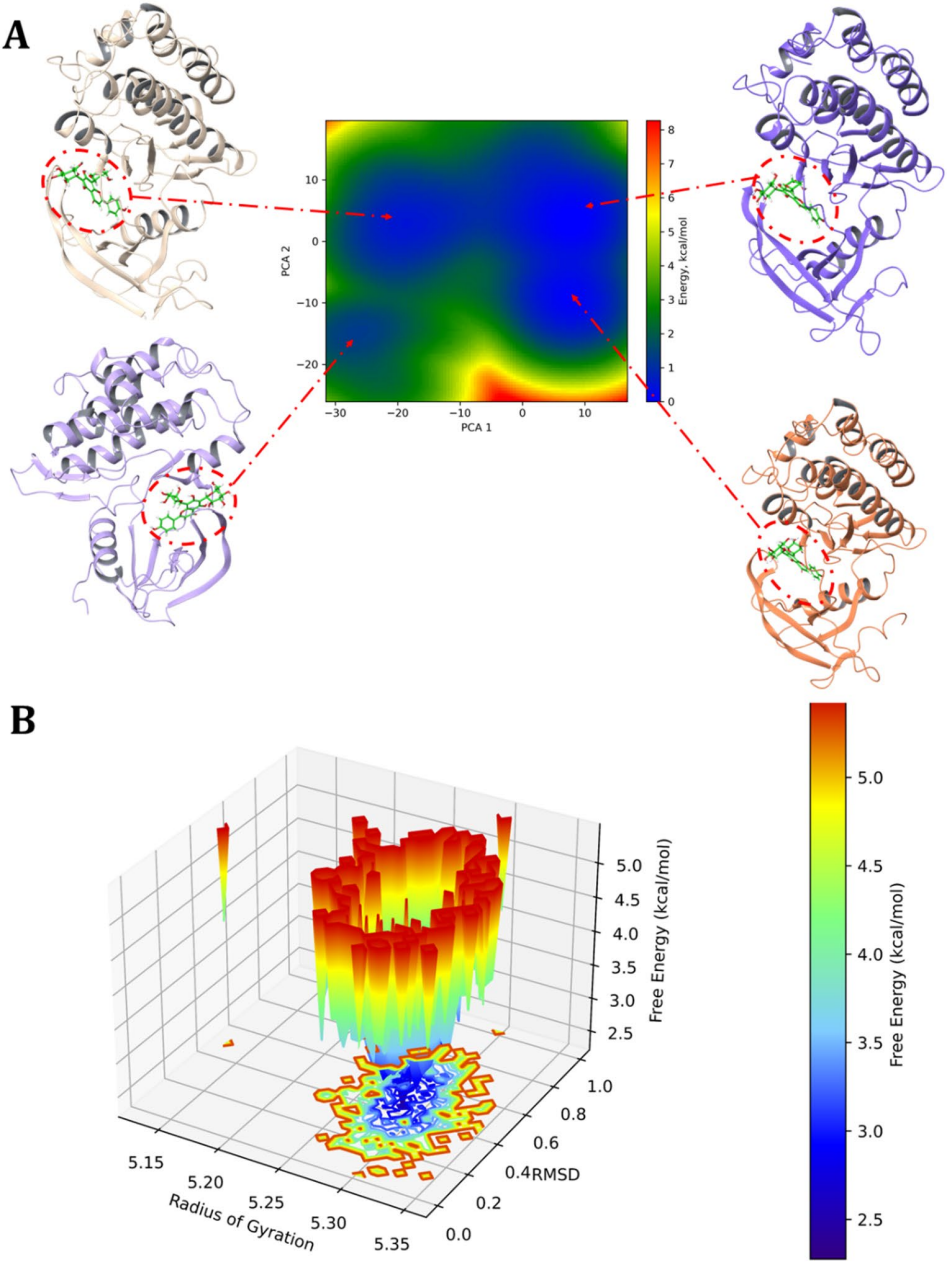
Low-energy conformational snapshots of the EGFR-neoschaftoside mapped onto the FEL contour plot derived from PCA (PC1 vs. PC2).

The resulting FEL plot revealed multiple energy basins, with the lowest energy minima (dark blue) located near the center of the PC1-PC2 space. These basins correspond to the most thermodynamically favorable conformational states sampled by the system. The presence of a dominant, well-defined global minimum, along with several shallow local minima, indicates that the EGFR-neoschaftoside complex adopts a relatively stable conformation, with limited transitions between metastable states. This energy landscape topology is consistent with the low RMSD fluctuations observed in both the protein and ligand, suggesting a restricted and energetically favorable binding mode. The smooth energy gradient surrounding the minima, transitioning from blue (low energy) to red (high energy), further suggests that major conformational rearrangements are energetically disfavored, and that the system remains confined within a narrow dynamic ensemble throughout the simulation (Fig. 16). This is complimenting to the residue interaction map, which identified persistent and dominant contacts with (VAL724, LYS875, and ARG841) that likely act as structural anchors limiting large-scale motion. Moreover, the PCA findings are supported by the DCCM matrix, where correlated motions were mostly localized within specific domains, and long range anti-correlations were present but did not disrupt the overall dynamics. This suggests that ligand binding helps stabilize certain shapes of EGFR and influences its internal movements without causing overall changes in the protein structure. Thus, the FEL derived from PCA confirms that the EGFR-neoschaftoside complex exhibits energetic and structural stability, characterized by a limited number of low-energy states. These results indicate that neoschaftoside acts as a stable binder throughout the 200 ns MD simulation.

## Discussion

Lung cancer is a complex and highly heterogeneous malignancy, primarily classified into two main types: NSCLC, which accounts for approximately 85% of cases, and SCLC, known for its aggressive nature and rapid progression^31^. This heterogeneity arises from diverse genetic mutations, epigenetic alterations, and environmental factors such as smoking, air pollution, and occupational exposure to carcinogens, all of which contribute to varied tumor behaviors and therapeutic responses^32^. The complexity of lung cancer presents significant challenges in drug development and personalized treatment^33^. In traditional medicine systems, plants and natural products have long been used to treat respiratory illnesses, and many modern anticancer agents have originated from such natural sources^18^. Natural small molecules, with their multi-target capabilities due to abundant hydrogen bond donors and acceptors, offer potential in modulating key signaling pathways involved in cancer^34^. However, their complex interactions make mechanistic elucidation difficult, a challenge now addressable through network pharmacology, which enables a systematic exploration of multi-target therapeutic actions at a systems biology level. As per the reported claims, *A. altissima* exhibits inhibitory effects on the invasive ability of cancer cells indicating its potential efficacy in cancer treatment^35^. Despite these promising findings, the regulatory mechanisms underlying effects on lung cancer are not elucidated. To address this, our study employed GO enrichment analysis, which suggested that *A. altissima* may exert its anticancer effects by influencing the phosphorylation and cascade reactions of transmembrane tyrosine kinases. The network diagram of bioactive compounds potential targets” was obtained through network pharmacology, which included 25 active compounds and 166 intersection targets of *A. altissima*. Among the 25 active compounds, neoschaftoside, rutin, 4 hydroxy benzoic acid and quassin have large degree value. Previous study on Rutin-loaded lipid-core nanoparticles (Rutin-LCNs) exhibited potent anti-cancer effects against non-small cell lung cancer (NSCLC) cells. They significantly inhibited cell proliferation and migration, as confirmed by MTT, trypan blue, scratch wound, and Boyden chamber assays^36^. 4-Hydroxybenzoic acid (4-HBA), another compound from *A. altissima*, selectively inhibits A549 lung cancer cell proliferation and induces pyroptosis by upregulating caspase-1, IL-1β, and IL-18^37^. Furthermore, neoschaftoside targeted EGFR and MAPK signaling pathways which were highly modulated in the KEGG enrichment analysis within the context of cancer pathways^38,39^. Predicted protein targets from SwissTargetPrediction are computational and may include false positives; thus, literature evidence and pathway enrichment analyses were integrated to enhance biological relevance and interpretative reliability. The differential expression analysis of hub genes using GEPIA2 revealed significant upregulation of key genes in LUAD and LUSC tumor tissues compared to normal controls, suggesting their involvement in lung cancer progression. Survival analysis further demonstrated that high expression levels of several hub genes, particularly EGFR, PIK3CA, PIK3CB, and PRKCA, are associated with poorer overall survival, indicating their prognostic relevance^40^. These genes are primarily involved in the EGFR and MAPK signaling pathways, highlighting their role in oncogenic signaling. Furthermore, the neoschaftoside displayed better binding affinity in the ligand binding domain of the EGFR TK pocket as compared to standard Osimertinib. To understand the dynamic motions of the neoschaftoside in the EGFR binding domain, a simulation of 200 ns was performed, which indicated stable RMSD, persistent protein-ligand contacts. Further DCCM and eigenvectors together revealed, the correlated motions between the residue present at the binding site of EGFR TK and neoschaftoside.

**Fig. 17:**
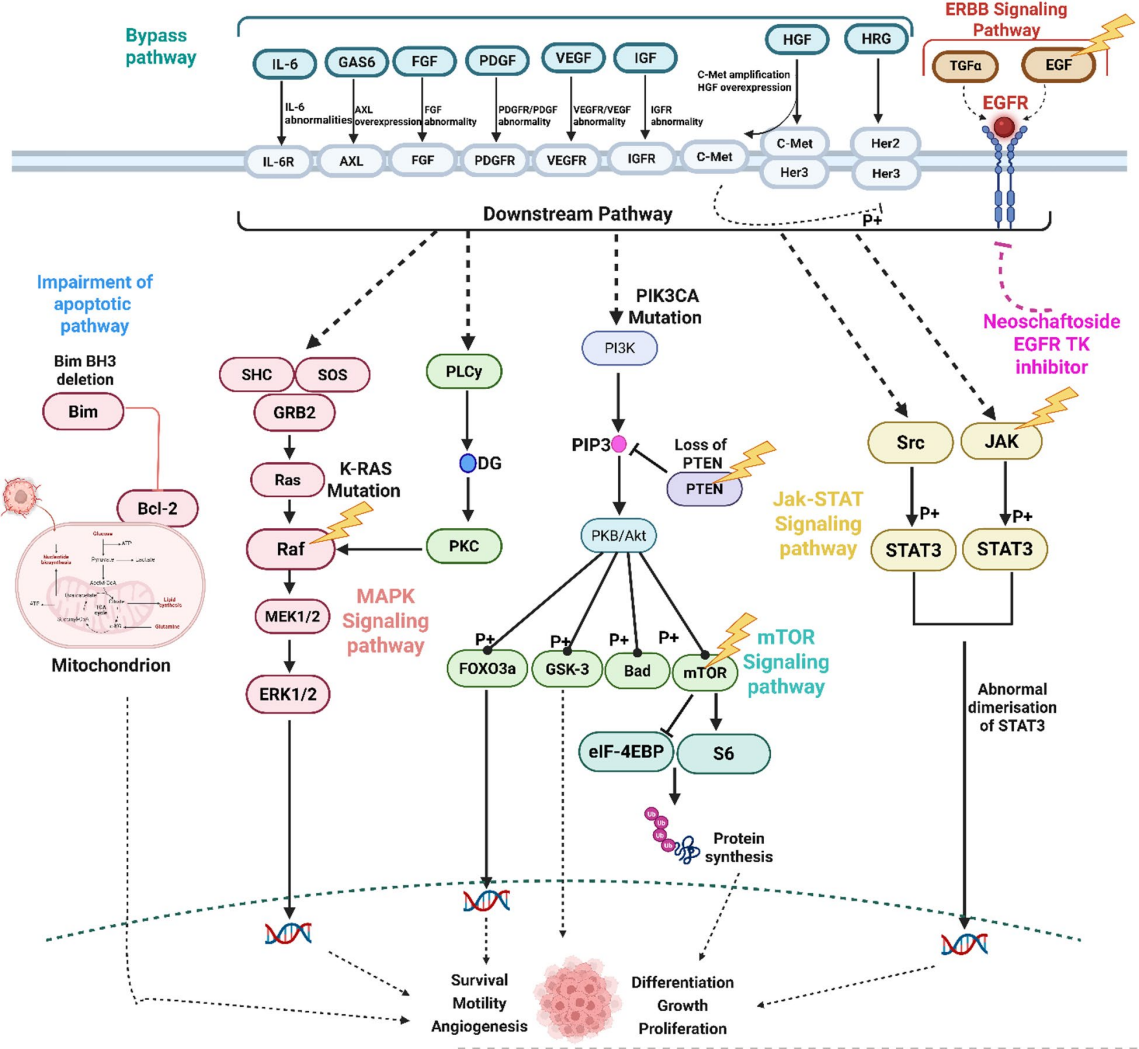
Protein checkpoints triggered by the neoschaftoside from *A. altissima* in pathways of cancer.

The findings indicate that neoschaftoside from *A. altissima* exhibits strong interaction potential, engaging with a broad spectrum of proteins associated with lung cancer, with EGFR emerging as a key target, via EGFR and MAPK signaling pathways (Fig. 17). Although these insights are primarily derived from computational analyses, which serve as valuable tools for preliminary screening, they may not fully recapitulate the complex biological responses observed in vivo. Therefore, further in depth experimental validation is required to elucidate the underlying signal transduction pathways and confirm the regulatory role of neoschaftoside through in vivo investigations.

## Conclusion

This study revealed that EGFR was modulated by the highest number of bioactive constituents from *A. altissima*, with neoschaftoside showing the greatest enrichment in protein interactions. Pathway enrichment analysis highlighted EGFR and MAPK signaling cascades as critical regulatory networks implicated in lung cancer progression. Consequently, the interaction between neoschaftoside and EGFR was further examined through computational approaches, demonstrating favorable binding affinity and stability. These findings provide a foundation for future research aimed at elucidating the regulatory influence of neoschaftoside on EGFR and MAPK associated proteins through in vitro and in vivo experimental validation.

## Supplementary Information

Below is the link to the electronic supplementary material.

Supplementary Material 1

Supplementary Material 2

Supplementary Material 3

## Data Availability

All the data that support the findings of this study are available within the article and supplementary tables.
